# The diagnosis and treatment of scrub typhus should be emphasized in non-endemic areas: A retrospective case series study

**DOI:** 10.1097/MD.0000000000032988

**Published:** 2023-02-22

**Authors:** Xin Song, Shu Xie, Xinhui Huang, Zhi Chen

**Affiliations:** a Medical Department of Nanchang University, Nanchang, Jiangxi, China; b Department of Emergency, Jiangxi Provincial People’s Hospital, Nanchang, Jiangxi, China; c The First Affiliated Hospital of Nanchang Medical College, Nanchang, Jiangxi, China.

**Keywords:** diagnosis, non-epidemic areas, scrub typhus

## Abstract

The morbidity of tsutsugamushi is increasing and is no longer limited to endemic areas. Delayed diagnosis and inappropriate treatment can cause severe complications and increase mortality rates. We conducted a retrospective case series of patients with scrub typhus at our institution to report our experience and discuss the diagnostic modalities. We encountered 21 cases of scrub typhus at our institution between 2014 and 2022. The average age of the patients was 52 years (range: 22–63 years), 11 (52%) were farmers, and 11 (52%) had clear outdoor activities. Twenty (95%) patients had an ineffective history of general antibiotic treatment. The classic presentation was repeated fever in 95% of patients. Seventeen patients (81%) had eschar mainly on the groin (35%) and armpit (35%). Common laboratory findings included eosinophilia (95%), elevated alanine aminotransferase (95%), elevated aspartate aminotransferase (86%), thrombocytopenia (76%), lower hemoglobin (71%), and neutrophilia (38%). Six (29%) patients received the treatment of tigecycline, 4 (19%) patients received the treatment of doxycycline, and 11 (52%) patients received the treatment of minocycline. After 3 days of specific treatment, the eosinophilic levels showed a recovery trend. Twenty (95%) patients fully recovered, and 1 (5%) died. Careful physical examination and medical history are important for the early diagnosis of scrub typhus; clinicians in non-endemic areas need to strengthen their understanding of scrub typhus.

## 1. Introduction

Tsutsugamushi is a febrile disease caused by rickettsia, also called “scrub typhus.”^[1]^ Other rickettsial infections and tsutsugamushi contribute to 25% to 50% of acute undifferentiated febrile illnesses in endemic regions.^[1]^ Approximately 1 billion people are threatened by scrub typhus yearly.^[2]^ An epidemiological survey 5 years ago showed that the median mortality rate of untreated patients was 6% and 1.4% for treated patients.^[3]^ Scrub typhus without appropriate treatment, can develop severe complications, such as multiple organ failure and disseminated intravascular coagulation, and the mortality rate can reach up to 70%.^[4]^ Recently, scrub typhus incidence has continued to increase globally because of global warming and changes in lifestyle^[4]^; it is no longer limited to the “tsutsugamushi triangle.”^[5]^ The early clinical symptoms of tsutsugamushi are nonspecific and include fever, chills, headache, vomiting, and myalgia.^[1]^ Before some characteristic signs are found, such as eschar or rash of the skin near the bite and swollen lymph nodes,^[2]^ the diagnosis of scrub typhus is difficult. Presently, this disease is still neglected by clinicians, especially in non-endemic areas.^[6–8]^ We conducted this retrospective analysis to summarize our diagnostic experience with scrub typhus and tried to emphasize this neglected febrile disease in non-endemic areas.

## 2. Methods

This study was approved by the Medical Ethics Committee of Jiangxi Provincial’s People’s Hospital (approval no. 2022-057). In this retrospective case series, we examined 21 patients with confirmed scrub typhus admitted to the Jiangxi Provincial’s People’s Hospital between November 2014 and September 2022. We reviewed their clinical data, including age, sex, region, occupation, admission to a physical examination, past medical history, test indicators, imaging examinations, treatment methods, and clinical outcomes.

### 2.1. Statistical analyses

All data were analyzed using the SPSS software (v23.0, IBM Corp., Armonk, NY). Data are presented as the mean ± standard deviation.

## 3. Results

### 3.1. Patients’ characteristic and clinical manifestations

We included 21 patients with scrub typhus at our institution for over 8 years, from 2014 to 2022. One case was observed in 2014, 1 in 2017, 5 in 2018, 3 in 2019, 3 in 2020, 2 in 2021, and 6 in 2022. Ten (48%) patients from Fuzhou city, 6 (29%) from Yichun city, 2 (10%) from Jiang city, 1 (5%) from Ganzhou city, and 1 (5%) from Shangrao city. The mean age of the patients was 52 years (range: 33–74 years); 14 (67%) were males; 11 (52%) were farmers, and 11 (52%) had clear outdoor activities. Twenty (95%) patients had an ineffective history of general antibiotic treatment. Six (29 %) patients had comorbidities; 1 had concurrent hypertension, diabetes, gastric ulcer, fatty liver, and gallstones (Table 1).

**Table 1:** Patient characteristics.

Patient characteristics	Mean ± SD or N
Age	52 **±** 11.29
Occupation	
Farmers	11
No farmers	10
Outdoor activity	
Go fishing	2
Weeding	3
Camping	1
Insect bite history	5
Comorbidity	
Alcoholic hepatitis	1
Viral hepatitis	1
Pulmonary tuberculosis	1
Hypertension	4
Clinical manifestations	
Repeated fever	20
Chills	9
Headache and vertigo	8
Digestive tract symptom	8
Fatigue	8
Muscular soreness	5
Chests pain and chest tightness	2
Characteristic signs	
Eschar	17
Lymphadenectasis	2
Multiple organ failure	5
Special examination	
NGS	5
Weil-Felix test	2
Treatment	
Tigecycline	6
Doxycycline	4
Minocycline	11
Length of stay (d)	
>20	2
10–20	7
<10	11
Outcomes	
Full recovery	20
Dead	1

NGS = next-generation sequencing.

Most patients had symptoms of repeated fever (95%), chills (45%), fatigue (40%), headache and vertigo (40%), and digestive tract symptoms (40%), including abdominal pain, abdominal distension, diarrhea, vomiting nausea, muscle soreness (20%), chest pain (10%), and chest tightness (5%). Among these patients, 4 (19%) had no eschar. In the other 17 (81%) patients, eschar was found on the groin (35%), armpit (35%), navel (6%), lower back (6%), buttocks (6%), perineum (6%), scrotum (6%), and dorsum of the foot (6%). Eleven (52%) patients’ eschars were found on admission. Lymph node enlargement near the skin bite was found in 2 patients (10%) during physical examination of the groin.

### 3.2. Characteristics of laboratory indicators (Table 2) and imaging examinations on admission

The complete laboratory data of 1 patient was lost, and we analyzed the data of the remaining 20 patients. The average of white blood cell level, neutrophils, eosinophils, erythrocyte, hemoglobin, platelet, prothrombin time, and activated partial thromboplastin time were 8.17 (3.57–31.72 × 10^9^/L), 68.5% (27.7–91.4%), 0.05% (0–0.2%), 4.03 (3.25–5.77 × 10^12^/L), 115.84 (85–160 g/L), 88 (27–316 × 10^9^/L), 13.12 (10.8–15.9 s), and 36.7 (24.1–52 s), respectively. Moreover, the average level of albumin, alanine transaminase, aspartate aminotransferase, creatinine, and uric acid were 29.77 (20.6–41.5 g/L), 104.53 (34–278 IU/L), 164.47 (36–597 IU/L), 124.82 (36–605 µmol/L), and 236.76 (54–602 µmol/L), respectively. All patients underwent imaging examinations at the hospital. In 12 (57%) patients, computed tomography (CT) showed patchy shadows and ribbon-like changes in both lungs and pleural effusion in 10 (48%). CT examination or ultrasonography suggested pericardial effusion in 7 (33%) patients and abdominal cavity and pelvic effusion in 7 (33%). Lymphadenopathy, including cervical, mediastinal, hilar, axillary, inguinal, and multiple small lymph nodes in the retroperitoneal and mesenteric spaces were observed 13 (62%), and 13 (62%) had splenomegaly, as indicated by CT tomography or ultrasonography.

**Table 2:** Laboratory results on admission.

Laboratory parameters (reference range)	Abnormal proportion (%)
Leukocyte count (3.5–9.5 × 10^9^/L)	10
Percentage of neutrophil (40–75%)	43
Percentage of eosinophils (0.4–8%)	95
Platelets (125–350 10^9^/L)	76
Hemoglobin (130–175 g/L)	71
PT (9.4–12.5 s)	67
APTT (25.4–38.4 s)	48
Creatinine (57–97 µmol/L)	14
Albumin (40–55 g/L)	95
Uric acid (170–420 µmol/L)	43
AST (15–40 IU/L)	86
ALT (9–50 IU/L)	95

ALT = alanine aminotransferase, APTT = activated partial thromboplastin time, AST = aspartate aminotransferase, PT = prothrombin time.

### 3.3. Diagnosis and treatment

Eighteen (86%) patients with scrub typhus were mainly diagnosed based on characteristic clinical manifestations. Seven (33%) patients were timely diagnosed with scrub typhus rely on eschar. Four (19%) patients had eschar on admission but were not diagnosed with scrub typhus promptly of the 5 patients who underwent next-generation sequencing (NGS) examined their blood sample, 3 cases were confirmed by NGS, and 2 performed NGS under the diagnosis of suspected scrub typhus. Another 2 took Weil-Felix test under the diagnosis of suspected scrub typhus. Most of our patients are not seriously ill, their symptoms soon improved after specific treatments, which may result in their low enthusiasm for standard diagnostic techniques. Six (29%) patients were treated with tigecycline (50 mg q12 hours, first dose 100 mg), 4 (19%) received doxycycline (100 mg q 12 hours), and 11 (52%) received minocycline (100 mg q 12 hours). Twelve (57%) patients had a hospitalization period of <10 days, and 2 (10%) stayed more than 20 days. Twenty (95%) patients fully recovered, and 1 (5%) died.

## 4. Discussion

Scrub typhus attacks mainly in autumn and winter^[1]^; it often occurs in the “tsutsugamushi triangle’’ in the Asia-Pacific area. However, with climate change and global warming, the incidence of scrub typhus in non-endemic areas is increasing. The tsutsugamushi disease in Jiangxi Province was mainly found in Ganzhou City and Fuzhou City. From 2006 to 2017, the annual reported incidence of scrub typhus was 0.79 per 100,000, and the incidence was on the rise.^[9]^ In our hospital, most scrub typhus patients come from Fuzhou city and Yichun city; since 2018, the diagnostic rate of scrub typhus in our hospital has increased significantly. But less than half of our patients were diagnosed with scrub typhus on admission. The diagnosis of scrub typhus cannot be overemphasized in non-endemic areas.

The diagnosis of scrub typhus is primarily based on clinical manifestations and examination.^[2]^ Mots scrub typhus patients are farmers or the elderly. These patients usually have a travel history or outdoor activities such as weeding, camping, fishing, and park walking in endemic areas.^[10]^ The clinical features of scrub typhus are usually nonspecific; because the pathological mechanism of scrub typhus is disseminated vasculitis. The clinical presentations can range from fever, chills, myalgia, meningitis, encephalitis, pneumonia, hepatitis, gastric ulcerations, and pancreatitis. Splenic infarction is rather rare.^[1]^ The characteristic signs of tsutsugamushi include eschar, rash, and swollen lymph nodes_._^[2]^ As the most important sign of tsutsugamushi, eschar can appear in 36.9% to 98.0% of patients_._^[11]^ The eschar tends to exist in some moist and hidden parts of the body, such as the armpit, groin, and scrotum.^[10]^ One case reported that an eschar was found in the external auditory canal.^[12]^ These indicates the importance of careful physical examination and medical history inquiry for clinicians to diagnose scrub typhus.

Laboratory diagnosis of scrub typhus requires immunological test. The Weil-Felix test was initially used to diagnose scrub typhus in resource-limited areas. However, due to its low sensitivity and specificity, most clinical settings no longer use it as a primary test.^[11]^ Rapid tests for detecting IgM antibodies against O. tsutsugamushi are far more superior to Weil Felix test, they are affordable and usable in remote resource poor settings, with results available in half an hour, these kits are reasonably specific and sensitive. While enzyme-linked immunosorbent assay has a sensitivity and specificity of 93% and 97.5%, respectively, adequate antibody levels are required for a positive response and early diagnosis.^[11]^ Polymerase chain reaction can prevent this problem; it can detect the infection from day 3, while it is often used under the premise of high clinical suspicion, such as characteristic eschar or rash.^[11]^ Recently, NGS methods have received increasing attention due to their high clinical diagnostic value, especially in infectious disease.^[13]^ However, such sophisticated laboratory tests in resource-limited settings are not feasible; sending samples to field facilities delays diagnosis simultaneously.^[11]^ In most case reports of scrub typhus diagnosed using NGS, it took at least 2 days to obtain results.^[14–16]^ NGS may be a helpful tool to clarify the source of infection in some cases, but ignoring basic clinical characteristics will lose the best time to intervene and, to some extent, may increase the unnecessary burden of the disease. In our study, most of the patients are not seriously ill, their symptoms soon improved after specific treatments, which may result in their low enthusiasm for standard diagnostic techniques.

**Figure 1.**
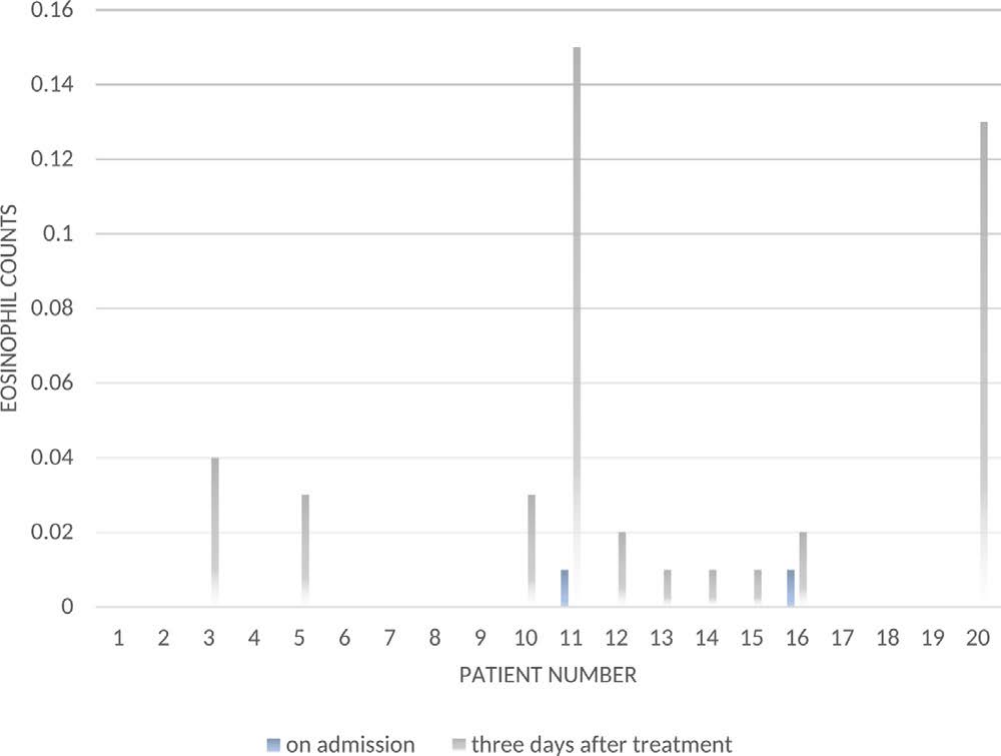
Eosinophil counts on admission and 3 days after treatment.

Laboratory indicators in patients with scrub typhus were mainly normal or low leukocyte and neutrophil levels. Patients with more severe conditions have lower platelet and albumin levels, implying a more severe state of illness, and elevated transaminase or creatinine levels indicate the extent of hepatic and renal function impairment.^[17]^ Interestingly, we indeed found that our patients had a low eosinophil count on admission. After specific treatment, the eosinophilic levels of most patients showed a recovery trend (Fig. 1). However, previous literature has hardly mentioned changes in eosinophils. Further studies are needed to explore the relationship between scrub typhus and eosinophil counts.

The preferred treatment for tsutsugamushi is tetracycline, which is recommended for 7 to 10 days. Limited by the drug resources of local hospital and the different clinical treatment experience of different clinicians, all patients cannot receive the same treatment. There are also many literature reports that the standard treatment regimen including doxycycline, azithromycin, rifampicin or chloramphenicol, which has no significant difference in treatment effect compared with tetracycline.^[18,19]^ Although all of our patients received appropriate treatment after admission, 1 with multiple underlying diseases developed severe multiple bleeding. Even with stronger antibiotics treatment, septic shock progressed, eventually leading to death. More attention should be paid to the early diagnosis of patients with scrub typhus, especially in non-endemic areas.

Our study had some limitations, including the retrospective nature of the study and the small number of patients. However, we do attach weight to its clinical implications in view of this underappreciated febrile illness, especially in non-endemic areas.

## 5. Conclusion

In conclusion, in non-endemic areas, the incidence trend of scrub typhus was not negligible. Early diagnosis of scrub typhus relies more on clinical experience, which requires clinicians to have a sufficient understanding of scrub typhus. Careful physical examination and medical history inquiry, synthesizing existing clinical auxiliary examination results, can help in a timely and accurate diagnosis of this disease.

## Acknowledgements

The authors would like to thank all the patients and doctors and nurses participated in this study.

## Author contributions

**Conceptualization:** Xin Song, Shu Xie, Xinhui Huang, Zhi Chen.

**Data curation:** Xin Song, Shu Xie, Xinhui Huang.

**Investigation:** Xin Song, Shu Xie, Xinhui Huang.

**Writing – original draft:** Xin Song, Shu Xie, Xinhui Huang.

**Writing – review & editing:** Xin Song, Shu Xie, Xinhui Huang, Zhi Chen.
